# Molecular Detection and Clinical Impact of *Helicobacter pylori* Virulence Genes in Gastric Diseases: A Study in Arequipa, Peru

**DOI:** 10.3390/biomedicines13040914

**Published:** 2025-04-09

**Authors:** Yuma Ita-Balta, Alice Zegarra-Adanaque, Johany Sanchez-Guillen, Miguel Farfán-Delgado, Carlos Ortiz-Castro, Alexis Germán Murillo Carrasco, Alejandro Miranda Pinto, Cecilia Manrique-Sam

**Affiliations:** 1 Escuela de Postgrado, Universidad Católica de Santa María, Arequipa 04013, Peru; yuma.ita@ucsm.edu.pe; 2 Escuela de Medicina Humana, Facultad de Medicina Humana, Universidad Católica de Santa María (UCSM), Arequipa 04013, Peru; azegarraa@ucsm.edu.pe (A.Z.-A.); jsanchezg@ucsm.edu.pe (J.S.-G.); mfarfan@ucsm.edu.pe (M.F.-D.); amiranda@ucsm.edu.pe (A.M.P.); mcmanriques1@hotmail.com (C.M.-S.); 3 Clínica Arequipa, Arequipa 04013, Peru; ortiz_san@hotmail.com; 4 Immunology and Cancer Research Group (IMMUCA), OMICS, Lima 15001, Peru

**Keywords:** *Helicobacter pylori*, virulence genes, gastric diseases, molecular detection, Peru, histopathology

## Abstract

**Background:** *Helicobacter pylori* is a globally prevalent pathogen and a major contributor to gastric diseases, including chronic gastritis, peptic ulcer disease, and gastric cancer. This study investigates the prevalence, distribution, and clinical relevance of its key virulence genes, *vacA* and *cagA*, in a Peruvian patient cohort. **Materials and Methods**: Fifty-one gastric biopsies were collected from patients with a presumptive diagnosis of *H. pylori*-induced gastritis at Hospital Carlos Alberto Seguín Escobedo in Arequipa, Peru, in March 2024. Two biopsies per patient—one from the antrum and one from the gastric body—were obtained during endoscopy. DNA extraction was performed using the Quick-DNA Fungal/Bacterial Kit (Zymo Research, USA). Molecular identification of *H. pylori* was conducted via PCR targeting the *glmM* gene, while the *vacA* and *cagA* virulence genes were detected using specific primers. Statistical analyses, including Pearson’s chi-square and Mann–Whitney tests, were applied to assess associations between virulence gene presence and clinical or histopathological variables. **Results**: Among the gastric biopsies, the *vacA* gene was detected in 37.3% of samples, while *cagA* was present in 17.6%. Statistical analysis revealed significant associations between *vacA* and specific clinical and endoscopic features, including erythematous gastropathy, nodular gastritis, and emetic syndrome, suggesting its localized role in disease pathogenesis. Additionally, the presence of *cagA* was significantly linked to moderate inflammatory intensity in gastric body biopsies, indicating its association with more severe histopathological outcomes. Chronic gastritis was the most common histopathological finding, with moderate intensity correlating strongly with the presence of virulence genes. **Conclusions**: These findings highlight substantial regional variability in the distribution and pathogenicity of *H. pylori* genotypes. This study underscores the importance of incorporating molecular diagnostics into routine clinical practice to improve diagnostic accuracy and inform region-specific therapeutic strategies. This is particularly crucial in endemic regions like Peru, where unique environmental and genetic factors may influence infection dynamics and disease outcomes.

## 1. Introduction

*Helicobacter pylori* (*H. pylori*, HP) is a Gram-negative, spiral-shaped bacterium that infects over 50% of the global population [1,2]. It is primarily acquired through oral transmission and colonizes the gastric mucosa after ingestion. Once established, the infection can persist indefinitely without treatment, leading to a range of gastroduodenal disorders, including chronic gastritis, gastric and duodenal ulcers, gastric cancer, and mucosa-associated lymphoid tissue (MALT) lymphoma [3].

*H. pylori* is a major cause of peptic ulcer disease and a key independent risk factor for distal gastric cancer. Notably, it was the first bacterium classified as a carcinogen by the International Agency for Research on Cancer (IARC) [4]. Gastric cancer continues to be a significant public health concern, ranking as the fifth most prevalent malignancy and the third leading cause of cancer-related deaths globally [5].

The pathogenicity of *H. pylori* is largely attributed to its virulence factors, with the most well studied being the *vacA* and *cagA* genes. The *cagA* gene encodes an oncoprotein, which is the best-characterized virulence marker and is strongly associated with an increased risk of peptic ulceration and gastric carcinoma [6]. The *vacA* gene, on the other hand, produces a multifunctional toxin that induces vacuolation and membrane channel formation in host cells, playing a key role in immune evasion and tissue damage [7].

The clinical outcomes of *H. pylori* infection are influenced by multiple factors, including the host’s immune response, the duration of infection, and environmental influences. Additionally, *H. pylori* exhibits genetic heterogeneity, with notable variability in virulence-associated genes such as *vacA* and *cagA*. This genetic diversity contributes to the observed geographical differences in the prevalence and severity of *H. pylori*-related diseases [7].

Although previous studies have explored the prevalence of *H. pylori* and its association with gastroduodenal diseases in various regions [1,2,3,4,5,7,8,9,10], there are significant gaps in understanding how specific virulence genes correlate with clinical and pathological outcomes, particularly in populations with unique environmental and genetic backgrounds. This is especially relevant in regions like Peru, where variations in altitude, dietary habits, and healthcare access may influence the pathogenic potential of *H. pylori*.

The current study aims to investigate the association of *H. pylori* virulence genes (*vacA* and *cagA*) with clinical and histopathological factors in a cohort of patients from Arequipa, Peru. By identifying patterns of virulence and their clinical impact, this research seeks to contribute to the growing body of knowledge on the role of *H. pylori* genotypes in disease pathogenesis, offering insights that may guide region-specific diagnostic and therapeutic strategies.

## 2. Materials and Methods

### 2.1. Sample Collection

Fifty-one samples were collected from patients with a presumptive diagnosis of HP-induced gastritis who attended the gastroenterology service at Hospital Carlos Alberto Seguín Escobedo (https://g.co/kgs/sishaXz, accessed on 10 March 2025) in Arequipa. Two additional gastric mucosa samples were obtained via endoscopy, one from the antrum and another from the gastric fundus (body). Clinical and histopathological data were retrieved from patient medical records.

### 2.2. Ethics Approval and Informed Consent

All participants provided voluntary informed consent. This study was approved by Favorable Opinion 021-2023 (Universidad Católica Santa María-UCSM) and IRB Approval No. 047-CIEI-UCID-GRAAR-ESSALUD-2023 (Peruvian Social Health Insurance—ESSALUD AREQUIPA).

### 2.3. Sample Size Analysis

Our sampling represented a significant proportion of patients treated during the study period (March 2024). The hospital performs approximately 8 endoscopies daily, amounting to 160 procedures monthly. Of these, we included 51 patients, representing 31.9% of the estimated total. Based on previous studies in Lima, Peru, reporting an HP prevalence of at least 38.54% [8,9,10], with *vacA* and *cagA* virulence genes present in 94% of strains, the OpenEpi software v.3.01 (https://www.openepi.com/, accessed on 20 November 2023) calculated that 50 samples were required to achieve 85% statistical power (confidence limit: 97%).

Each patient provided 2 biopsies (1 from the antrum and 1 from the gastric body), resulting in 102 samples being analyzed in this study.

### 2.4. Clinical Information

The infection duration was estimated using a structured questionnaire addressing symptom duration and relevant medical history. Endoscopy was performed as part of the initial diagnostic evaluation based on clinical suspicion of HP infection, including symptoms such as burning abdominal pain and dyspepsia. Biopsies were collected from the antrum and gastric body during the procedure for subsequent analysis. The presence of HP was confirmed by direct microscopy of paraffin-embedded biopsies, identifying spiral bacteria in histological sections stained accordingly.

### 2.5. DNA Extraction

Samples were placed in Eppendorf tubes containing 5% phenol. DNA was extracted using the Quick-DNA Fungal/Bacterial Kit (Zymo Research, Irvine, CA, USA), following the manufacturer’s recommendations. DNA quality and concentration were assessed using an Implen NanoPhotometer™ NP80 spectrophotometer at 260 and 280 nm. Only samples with a 260/280 ratio between 1.6 and 1.9 were processed further.

### 2.6. Molecular Identification of HP

The *glmM* gene was selected as the target for molecular identification using polymerase chain reaction (PCR). Specific primers were designed de novo using Primer3 software (https://www.primer3plus.com; accessed on 20 October 2023) based on the reference sequence ID 93236447 (https://www.ncbi.nlm.nih.gov/gene/?term=93236447; accessed on 20 October 2023). Primer characteristics, including melting temperature, GC content, and potential secondary structures (e.g., dimers), were thoroughly evaluated. The following primers were synthesized: Forward 5′-CCATGCACGATATTCCCTAA-3′ and Reverse 5′-GATAGACGATGTGATAGGGC-3′. The expected amplicon size was 359 bp. To confirm the specific amplification of the target region, representative PCR products were subjected to Sanger sequencing by Macrogen (Seoul, Republic of Korea). The resulting electropherograms are provided in Supplementary Source S1.

Amplification was performed using PowerPol 2X PCR mix with Dye (Abclonal Science, Alameda, CA, USA) under the following conditions: initial denaturation at 94 °C for 5 min, 35 cycles of 94 °C for 30 s, 58 °C for 30 s, and 72 °C for 30 s, followed by a final extension at 72 °C for 10 min. PCR products were resolved by 2% agarose gel electrophoresis at 90 V for 30 min in 1X TAE buffer, with amplicon size confirmed using a 100 bp marker.

### 2.7. Virulence Gene Analysis

The prevalence of *vacA* and *cagA* virulence genes was determined using the following primers:

*vacA* [11]: Forward 5′-CACAGCCACTTTCAATAACGA-3′, Reverse 5′-GTCAAAATAATTCCAAGGG-3′.

*cagA* [12]: Forward 5′-TTGACCAACAACCACAAACCGAAG-3′, Reverse 5′-CTTCCCTTAATTGCGAGATTC-3′.

PCR products were evaluated by 2% agarose gel electrophoresis at 90 V for 40 min. Amplicon size was confirmed using a 100 bp marker.

### 2.8. Statistical Analysis

Statistical analyses were conducted to correlate clinical and histopathological factors with *vacA* and *cagA* genes. Pearson’s chi-square test was used for categorical variables in SPSS v.25. The Mann–Whitney test was applied to quantitative variables after confirming a non-normal distribution. Boxplots illustrating key findings were generated using R v.4.4.0.

## 3. Results

### 3.1. Patient Characteristics

A total of 51 samples from patients with a suspected diagnosis of *H. pylori* were analyzed. The variables considered in this study included a prior history of *H. pylori* infection, endoscopic findings, clinical features, histopathological characteristics, pathological and molecular diagnosis of *H. pylori*, and the presence of the *glmM*, *vacA*, and *cagA* genes.

Table 1 provides a descriptive analysis of the demographic characteristics, clinical history, endoscopic findings, and clinical features from gastric samples of patients with suspected *H. pylori* infection. The majority of the population was male. Histopathological analysis revealed a marked prevalence of type I hiatal hernia (23.5%), antral erythematous gastropathy (56.9%), and body erythematous gastropathy (54.9%).

### 3.2. Pathological and Molecular Prevalence of H. pylori

Table 2 summarizes the histopathological characteristics and the pathological and molecular prevalence of *H. pylori* in gastric samples from patients with suspected infection. Figure S1 illustrates the detection of the *glmM*, *vacA*, and *cagA* genes in representative samples. Chronic gastritis was observed in 90% of the analyzed biopsies, with moderate intensity being predominant in both the antrum (25.5%) and the body (27.5%).

### 3.3. Association of H. pylori with Endoscopic Features

The results of the analysis are shown in Table 3, where a significant association (*p* < 0.001) was found between pathological diagnosis, molecular diagnosis, and the prevalence of the virulence gene *vacA*. A positive association was also observed with antral erythematous gastropathy, antral nodular chronic gastropathy, and chronic atrophic pangastropathy (*p* < 0.05). Regarding the *cagA* gene, a significant positive association was found between molecular diagnosis and moderate infectious activity in the gastric body (*p* < 0.05).

**Figure 1:**
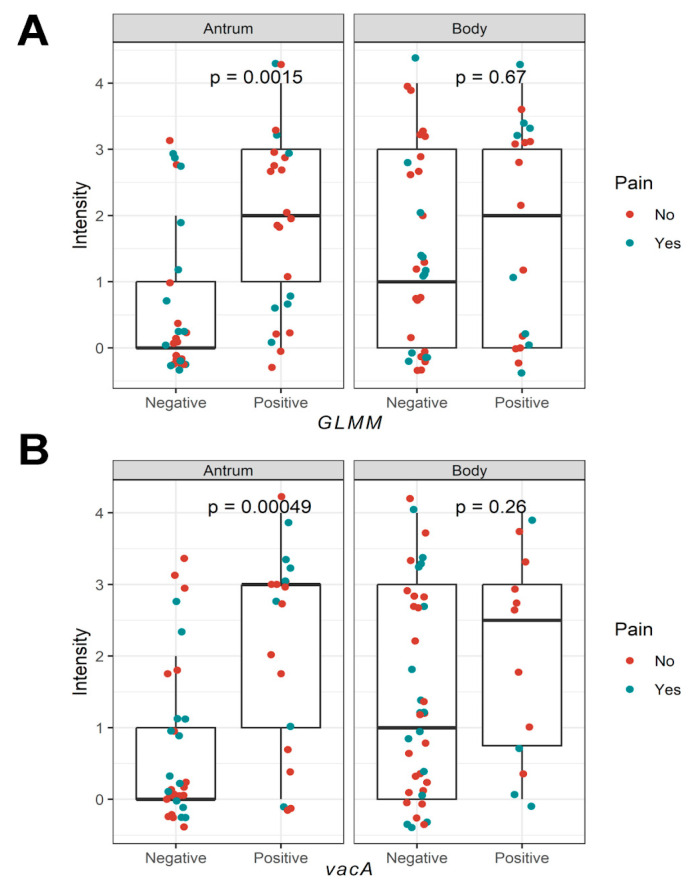
Comparison of intensity in gastric regions (antrum and body) based on positivity for different factors. (**A**) Analysis showing the *glmM* gene, demonstrating a significant difference in the antrum (*p* = 0.0015) but not in the body (*p* = 0.67). (**B**) Comparison based on the presence of the *vacA* gene reveals a significant difference in the antrum (*p* = 0.00049) but not in the body (*p* = 0.26). Dots represent individual values, with colors indicating the presence (blue) or absence (red) of pain. Statistical comparisons were performed using the Mann–Whitney test.

Quantitative evaluations of the molecular detection of *H. pylori* (via *glmM* gene) or its virulence genes (*vacA* or *cagA*) revealed that *H. pylori* (Figure 1A) and the *vacA* gene (Figure 1B) were associated with higher intensity levels in the gastric antrum (*p* = 0.0015 and *p* = 0.00049, Mann–Whitney test). However, this association was not significant when the infection was detected in the gastric body (*p* > 0.05). Similarly, no significant differences were observed in other quantitative variables such as age or duration of infection.

Based on the results presented in Table 3, 29.41% of the study population with a prior history of *H. pylori* infection carried the virulence gene *vacA*. However, no statistical relationship was found between prior infection and the prevalence of *vacA*.

The prevalence of *vacA* varied between diagnostic methods, with 37.3% of pathological diagnoses and 52.9% of molecular diagnoses identifying the gene. These findings suggest that at least half of the *H. pylori*-positive patients harbor bacterial strains with the *vacA* virulence gene and indicate the potential for false-negative results with pathological diagnosis.

A significant association was identified between the prevalence of the *vacA* gene and four endoscopic features: antral erythematous gastropathy, antral chronic nodular gastropathy, and antral ulcer classified as Forrest III. However, due to the limited number of cases with Forrest ulcers, additional samples are needed to confirm the relationship between *vacA* presence and these findings. No significant associations were observed for other endoscopic features. Clinically, a highly significant association (*p* < 0.001) was found between the presence of the *vacA* gene and emetic syndrome. Among the 17 cases presenting with emetic syndrome, 14 carried the *vacA* gene. No significant associations were detected for other clinical manifestations.

Regarding the *cagA* gene, the results presented in Table 3 indicate that 15.7% of the study population with a prior history of *H. pylori* infection harbored the *cagA* virulence gene. However, no statistical association was observed between prior infection and *cagA* prevalence. No significant relationship was found between pathological diagnosis and *cagA* prevalence. Although molecular testing showed a significant association, the high proportion of samples diagnosed as negative suggests potential issues related to detection processes or sample quality.

Unlike the associations observed between *vacA* and specific biopsy results, no significant correlations were found between *cagA* and the endoscopic findings. Additionally, *cagA* did not show any association with the clinical characteristics of patients with suspected *H. pylori* infection. For *cagA*, a significant relationship was observed only in gastric body biopsies, where moderate staining intensity was most frequently associated with *cagA* prevalence.

### 3.4. Histopathological Analysis of the Antrum

A strong relationship was observed between chronic gastritis and the prevalence of the *vacA* gene. A moderate intensity of inflammation was particularly associated with *vacA* presence. Additionally, a significant relationship was found between *H. pylori* activity and biopsy analysis, although no relationship was detected between *vacA* and erosiveness.

### 3.5. Histopathological Analysis of the Body

Chronic gastritis was present in all samples, with 52.9% of strains carrying the *vacA* gene. A significant relationship was found between inflammation intensity and *vacA* prevalence, with moderate intensity being most associated. A highly significant relationship was also observed between *H. pylori* activity and biopsy analysis, but no relationship was found between vacA and erosiveness.

## 4. Discussion

*Helicobacter pylori* is a globally prevalent pathogen and a well-established etiological agent of chronic gastritis, peptic ulcer disease, and gastric cancer [13,14]. While most infections lead to mild chronic gastritis, approximately 1–2% progress to gastric cancer, particularly in individuals harboring *cagA*-positive strains [15]. In this study, the *vacA* gene was identified in 37.3% of antral samples and 23.5% of gastric body samples, while the *cagA* gene was present in 17.6% of samples from both regions. These rates are significantly lower than those reported by Oliveira et al. in Brazil, where *cagA* prevalence reached 80.5% [16]. Similarly, Kishk et al. documented a *vacA* prevalence of 61.6% in Egypt, with higher infection rates in older individuals and a strong association with dyspepsia [17]. However, our data did not show a significant relationship between dyspepsia and vacA prevalence, suggesting potential regional differences in bacterial strains or host–pathogen interactions.

This study expands knowledge about *H. pylori* virulence in Latin America by providing specific evidence from Arequipa, Peru, a high-altitude region (2335 m) with unique environmental and genetic characteristics [18,19]. Compared to previous studies in Lima, our findings highlight significant differences in the prevalence of virulence genes and their clinical associations. Notably, the low *cagA* prevalence (17.6%) contrasts with reports from Colombia [20], suggesting regional genetic divergence among *H. pylori* strains, potentially influenced by ecological and host-related factors. These differences emphasize the need for local strain genotyping to inform public health strategies and clinical guidelines tailored to specific populations.

From a mechanistic perspective, *H. pylori* has evolved to survive in the harsh gastric environment through various virulence factors [21]. Among them, *vacA* and *cagA* play critical roles in pathogenesis [22]. The *cagA* gene encodes the CagA oncoprotein, which is translocated into gastric epithelial cells via a type IV secretion system. Once inside the host cell, CagA undergoes tyrosine phosphorylation and disrupts multiple signaling pathways, including SHP-2, ERK, and NF-κB, leading to cell proliferation, inflammation, and oncogenic potential [23,24,25]. On the other hand, *vacA* encodes a vacuolating cytotoxin that induces host cell vacuolation, mitochondrial dysfunction, and immune evasion by interfering with T-cell activation and antigen presentation [26]. These mechanisms explain the distinct clinical and histopathological associations observed for both genes in our study.

Our results indicate preferential *H. pylori* colonization of the gastric antrum, which can be attributed to its higher density of adhesion receptors, such as BabA and SabA [8,20]. These adhesins facilitate efficient bacterial attachment and persistence, leading to localized inflammation through the release of pro-inflammatory cytokines [8]. Additionally, we observed a significant association between *vacA* and specific clinical and endoscopic features, such as emetic syndrome, erythematous gastropathy, and nodular gastritis. In contrast, *cagA* was more strongly correlated with moderate histopathological intensity in gastric body biopsies, underscoring its role in tissue damage and disease progression. Globally, approximately 50–70% of all *H. pylori* strains express *cagA*, often in combination with other virulence factors, including *vacA* [27]. However, the lower *cagA* prevalence observed in our population may reflect regional strain variability, which could limit its diagnostic utility.

Recent studies have also documented the presence of *cagA*, *vacA*, and *hrgA* genes in *H. pylori* strains isolated from poultry meat in Egypt, with prevalences of 77.8%, 66.7%, and 100%, respectively [28]. These findings suggest not only a high virulence burden in zoonotic strains but also potential foodborne transmission risks. In contrast, our findings indicate lower frequencies of these genes in Peruvian human strains, which may be linked to local adaptations or differences in bacterial reservoirs.

Despite the contribution to its field, this study has some limitations that should be considered. First, the hospital-based sampling may introduce selection bias by primarily including patients with evident gastrointestinal symptoms and potentially excluding asymptomatic carriers. Future research should include multicenter cohort studies to address this geographic heterogeneity. Additionally, longitudinal studies tracking disease progression in patients with specific virulence profiles could provide insights into the long-term impact of *H. pylori* infection. Regarding the number of evaluated genes, targeted technologies (PCR) are useful for characterizing the sample of this study. Nevertheless, the whole-genome sequencing (WGS) of Peruvian *H. pylori* strains could uncover novel virulence or host adaptation mechanisms, further expanding our understanding of the bacterium’s pathogenic potential.

Overall, this study provides key insights into the epidemiology of *H. pylori* in Arequipa, Peru, highlighting significant regional variability in virulence gene prevalence and clinical associations. A strong correlation was observed between *vacA* and endoscopic features such as antral erythematous gastropathy, nodular antral gastritis, and Forrest III antral ulcers. Clinically, a highly significant relationship (*p* < 0.001) was found between *vacA* and the occurrence of emetic syndrome, as well as a strong correlation with chronic gastritis. Regarding *cagA*, its prevalence was significantly associated only with histopathological severity in gastric body biopsies, with moderate-intensity biopsies showing the highest *cagA* prevalence.

## 5. Conclusions

This study provides valuable insight into the understanding of *Helicobacter pylori* pathogenesis in a region of Peru that has been less described (Arequipa), emphasizing the significant role of virulence factors in gastric disease and the marked regional differences in their prevalence and clinical associations. Endoscopic characteristics showed a moderate to high statistical association between the presence of *vacA* and conditions such as antral erythematous gastropathy, nodular chronic gastropathy of the antrum, and Forrest III antral ulcers. Clinically, a highly significant relationship (*p* < 0.001) was observed between the presence of the *vacA* gene and the occurrence of emetic syndrome, as well as a strong correlation between *vacA* prevalence and chronic gastritis. Regarding the *cagA* gene, its prevalence was significantly associated only with the pathological intensity observed in body biopsies. Among these, biopsies with moderate pathological intensity exhibited the highest prevalence of the *cagA* gene.

These findings reinforce the differential roles of *vacA* and *cagA* genes in *H. pylori*-related diseases. While *vacA* shows stronger associations with specific clinical and endoscopic features, *cagA* is more prominently linked to histopathological severity. This study underscores the necessity of integrating molecular diagnostics into clinical protocols and further investigating the mechanisms underlying these associations to improve disease management and treatment strategies tailored to regional and individual patient profiles. Future investigations should focus on longitudinal cohort studies to assess how virulence genotypes correlate with disease progression. Additionally, applying WGS to *H. pylori* strains from different regions of Peru could help identify not only classic virulence genes such as *cagA* and *vacA* but also other genetic elements associated with pathogenesis and antimicrobial resistance. This approach would contribute to refining predictive models and improving therapeutic decision-making in *H. pylori* management.

## Figures and Tables

**Table 1:** Population characteristics, clinical history, endoscopic, and clinical features.

Item	Number (N = 51)	Percentage
**Population Characteristics**		
**Sex**		
Female	33	64.7%
Male	18	35.3%
**History of HP infection**		
Duration of Infection (months)	7.04	
Previous HP Infection		
Yes	23	45.1%
No	28	54.9%
**Endoscopic Features**		
Hiatal Hernia		
HH1	12	23.5%
HH2	8	15.7%
HH3	1	2.0%
**Gastropathies**		
Chronic Gastritis (CG)	1	2.0%
Chronic Gastritis of Antrum (CGA)	5	9.8%
Erythematous Gastritis of Antrum (EGA)	29	56.9%
Erythematous Gastritis of Body (EGC)	28	54.9%
Nodular Chronic Gastritis of Antrum (NCGA)	5	9.8%
Chronic Pan-Gastritis (CPG)	1	2.0%
Atrophic Pan-Gastritis (APG)	1	2.0%
Erosive Pan-Gastritis (EPG)	1	2.0%
**Other Conditions**		
Gastric Ulcers (GU)	1	2.0%
Mallory–Weiss Syndrome (MWS)	1	2.0%
Mild Esophagitis (ME)	2	3.9%
Atrophic Gastritis of Body (AGB)	1	2.0%
Forrest III Antral Ulcer (FAU3)	1	2.0%
**Clinical Features**		
Dyspepsia	29	56.9%
Emetic Syndrome	17	33.3%
Abdominal Pain	20	39.2%

**Legend:** Hiatal Hernia I (HH1), Hiatal Hernia II (HH2), Hiatal Hernia III (HH3); Chronic Gastritis (CG), Chronic Gastritis of Antrum (CGA), Erythematous Gastritis of Antrum (EGA), Erythematous Gastritis of Body (EGC), Nodular Chronic Gastritis of Antrum (NCGA), Chronic Pan-Gastritis (CPG), Atrophic Pan-Gastritis (APG), Erosive Pan-Gastritis (EPG); Gastric Ulcers (GU), Mallory-Weiss Syndrome (MWS), Mild Esophagitis (ME), Atrophic Gastritis of Body (AGB), Forrest III Antral Ulcer (FAU3).

**Table 2:** Descriptive analysis of anatomopathological characteristics and pathological and molecular prevalence of HP.

Item	Number (N = 51)	Percentage
**Anatomopathological Features**		
**Antral Pathology**		
Chronic Gastritis	46	90.2%
**Intensity**		
Mild	7	13.7%
Superficial	5	9.8%
Moderate	13	25.5%
Severe	2	3.9%
Erosivity	15	29.4%
Activity	17	33.4%
HP Presence	18	35.3%
**Body Pathology**		
Chronic Gastritis	51	100.0%
**Intensity**		
Mild	12	23.5%
Superficial	3	5.9%
Moderate	14	27.5%
Severe	5	9.8%
Erosivity	5	9.8%
Activity	19	37.2%
HP Presence	19	37.3%
**HP Pathological Diagnosis**		
Positive	20	39.2%
Negative	31	60.8%
**HP Molecular Diagnosis**		
Positive	36	70.6%
Negative	15	29.4%
**Genes in Antrum**		
*glmM*	20	39.2%
*vacA*	19	37.3%
*cagA*	9	17.6%
**Genes in Body**		
*glmM*	34	66.7%
*vacA*	12	23.5%
*cagA*	9	17.6%

**Table 3:** Association between previous HP infection history, endoscopic, clinical, and anatomopathological characteristics, and the prevalence of HP virulence genes.

Item	*vacA*			*cagA*		
	N (%)	Chi2	*p*-Value	N (%)	Chi2	*p*-Value
**History of HP Infection**						
Prior HP Infection	15 (29.41%)	0.010	0.921	8 (15.7%)	0.39	0.843
Pathological Diagnosis of HP						
Positive	19 (37.3%)	23.362	<0.001 ***	7 (13.7%)	0.942	0.332
Negative	8 (15.7%)	-	-	7 (13.7%)	-	-
**Molecular Diagnosis**						
Positive	27 (52.9%)	23.906	<0.001 ***	13 (25.5%)	4.609	0.032 *
Negative	9 (17.6%)	-	-	23 (45.1%)	-	-
**Endoscopic Characteristics**						
Hiatal Hernia						
HH1	7 (13.7%)	4.211	0.755	3 (5.9%)	9.274	0.234
HH2	4 (7.8%)	-	-	2 (3.9%)	-	-
HH3	1 (1.9%)	-	-	1 (1.9%)	-	-
Gastropathy						
GC	1 (1.9%)	4.211	0.755	1 (1.9%)	9.274	0.234
GCA	0	-	-	2 (3.9%)	4.251	0.514
GEA	14 (27.5%)	13.505	0.019 **	7 (13.7%)	-	-
GECu	16 (31.4%)	6.476	0.263	6 (11.8%)	4.487	0.482
GCNA	5 (9.8%)	13.505	0.019 **	2 (3.9%)	4.251	0.514
PC	1 (1.9%)	6.476	0.263	0	4.487	0.482
PCAT	0	13.505	0.019 **	1 (1.9%)	4.251	0.514
PE	1 (1.9%)	6.476	0.263	0	4.487	0.482
Others						
MUG	0	4.211	0.755	1 (1.9%)	9.274	0.234
SMW	1 (1.9%)	-	-	0	-	-
ELLA	1 (1.9%)	-	-	2 (3.9%)	-	-
GACu	1 (1.9%)	6.476	0.263	0	4.487	0.482
UAF3	1 (1.9%)	13.505	0.019 **	0	4.251	0.514
**Clinical Characteristics**						
Dyspepsia	15 (29.41%)	0.040	0.842	11 (21.6%)	3.708	0.054
Emetic Syndrome	14 (27.5%)	8.854	0.003 ***	4 (7.8%)	0.197	0.657
Abdominal Pain	10 (19.6%)	0.114	0.735	4 (7.8%)	0.917	0.338
**Anatomopathological Characteristics**						
Antrum Pathology						
Chronic Gastritis	27 (52.9%)	6.236	0.013 **	14 (27.5%)	2.098	0.148
Intensity						
Mild	3 (5.9%)	9.932	0.042 *	2 (3.9%)	4.021	0.403
Superficial	2 (3.9%)	-	-	3 (5.9%)	-	-
Moderate	11 (21.6%)	-	-	4 (7.8%)	-	-
Severe	2 (3.9%)	-	-	0	-	-
Erosivity	10 (19.6%)	1.607	0.205	5 (9.8%)	0.369	0.543
Activity	16 (31.4%)	17.405	<0.001 ***	6 (11.8%)	0.795	0.672
Presence of HP	17 (33.3%)	19.234	<0.001 ***	7 (13.7%)	1.827	0.176
Body Pathology						
Chronic Gastritis	27 (52.9%)	-	-	14 (27.5%)	-	-
Intensity						
Mild	5 (9.8%)	17.458	0.002 ***	1 (1.9%)	10.475	0.033 *
Superficial	1 (1.9%)	-	-	1 (1.9%)	-	-
Moderate	12 (23.5%)	-	-	8 (15.7%)	-	-
Severe	5 (9.8%)	-	-	0	-	-
Erosivity	4 (7.8%)	1.830	0.401	1 (1.9%)	0.406	0.816
Activity	18 (35.3%)	21.356	<0.001 ***	7 (13.7%)	1.722	0.423
Presence of HP	18 (35.3%)	21.560	<0.001 ***	7 (13.7%)	1.589	0.452

**Legend**: Hiatal Hernia I (HH1), Hiatal Hernia II (HH2), Hiatal Hernia III (HH3); Chronic Gastropathy (GC), Chronic Antrum Gastropathy (GCA), Erythematous Antrum Gastropathy (GEA), Erythematous Body Gastropathy (GECu), Nodular Chronic Antrum Gastropathy (GCNA), Chronic Pangastropathy (PC), Atrophic Chronic Pangastropathy (PCAT), Erosive Pangastropathy (PE). Gastric Ulcers (MUG), Mallory-Weiss Syndrome (SMW), Los Angeles Grade A Esophagitis (ELLA), Atrophic Body Gastritis (GACu), Antral Ulcer Forrest III (UAF3). * *p* < 0.05; ** *p* < 0.01; *** *p* < 0.001. Statistical analysis was performed using Pearson’s chi-square, SPSS25 (IBM, Armonk, NY, USA), significance level 95%.

## Data Availability

The original contributions presented in this study are included in the article/Supplementary Materials. Further inquiries can be directed to the corresponding author.

## Supplementary Materials

The following supporting information can be downloaded at https://www.mdpi.com/article/10.3390/biomedicines13040914/s1: Figure S1: Representative agarose gels showing the amplification of virulence genes of Helicobacter pylori. (A) Gene *vacA* in the antrum—positive for M3, M5, M6, and M10. (B) Gene *cagA* in antrum—positive for M1, M7, M10, M15, M16, and M17. (C) Gene *cagA* in the body—positive for M1, M2, M3, and M6. (D) Gene *vagA* in the body—positive for M9 and M12. In all cases, a 100pb ladder (PCRBIO Ladder IV, cat. PB40.14-01. PCR Bio, London, UK) was included in lanes labeled as “M” in red letters. Source S1: Electropherograms of the *glmM* gene obtained through Sanger sequencing in representative samples. The expected binding regions of the forward and reverse primers are highlighted in purple and pink, respectively.
