# Supplementary figures and images for: Molecular Detection and Clinical Impact of Helicobacter pylori Virulence Genes in Gastric Diseases: A Study in Arequipa, Peru
Source: Biomedicines. 2025 Apr 9;13(4):914. doi: 10.3390/biomedicines13040914 (PMC12025056; doi:10.3390/biomedicines13040914)

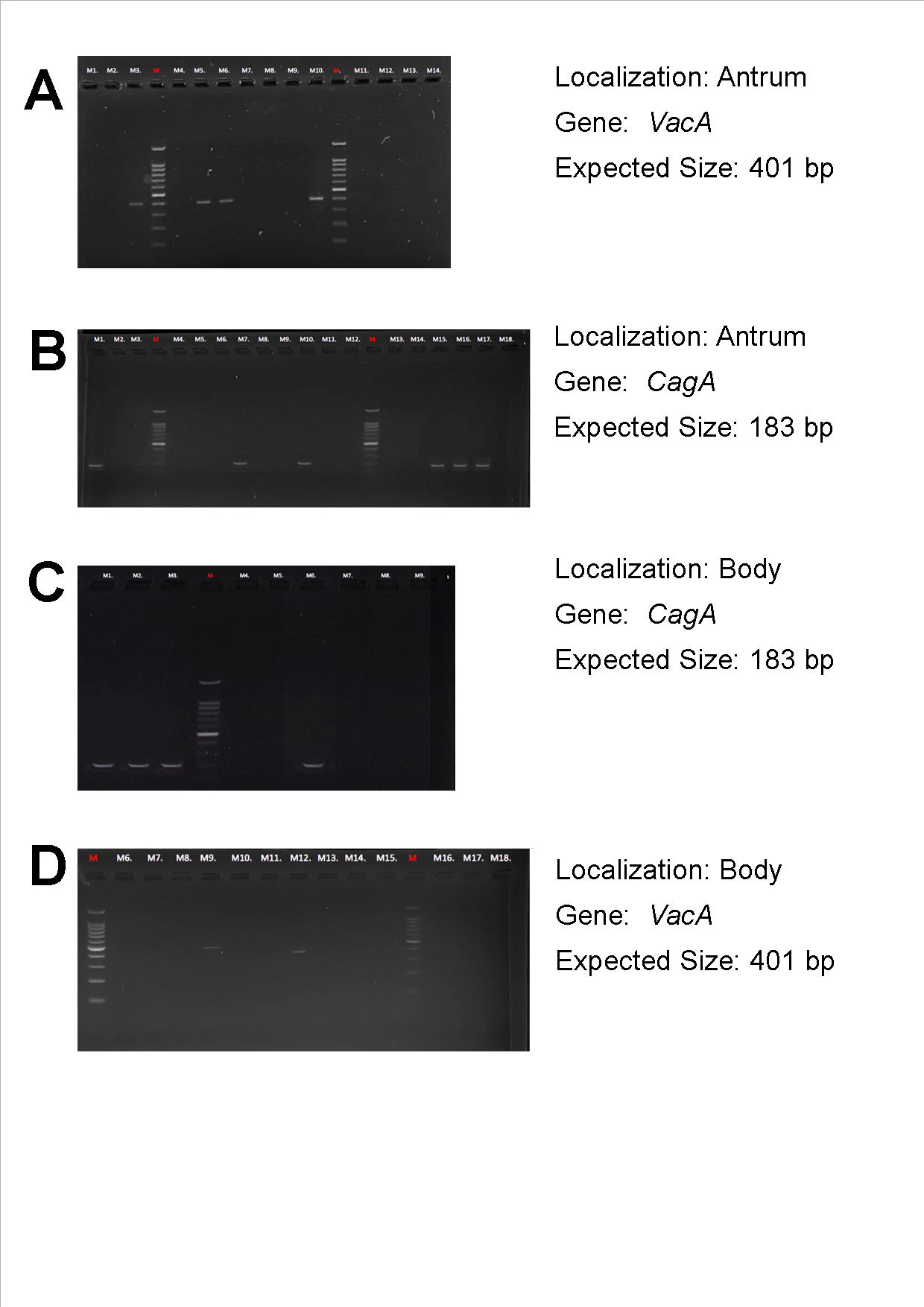

Supplement: Supplementary file 1 [file biomedicines-13-00914-s001.zip › Figure S1. Representative agarose gels showing amplification of virulence genes of Helicobacter pylori..png]

File: 34HP\_GLMM.ab1 Run Ended: 2024/10/3 22:36:41 Signal G:1512 A:2267 C:2750 T:3849  
Sample: 34HP\_GLMM Lane: 76 Base spacing: 13.890334 943 bases in 14569 scans Page 1 of 2

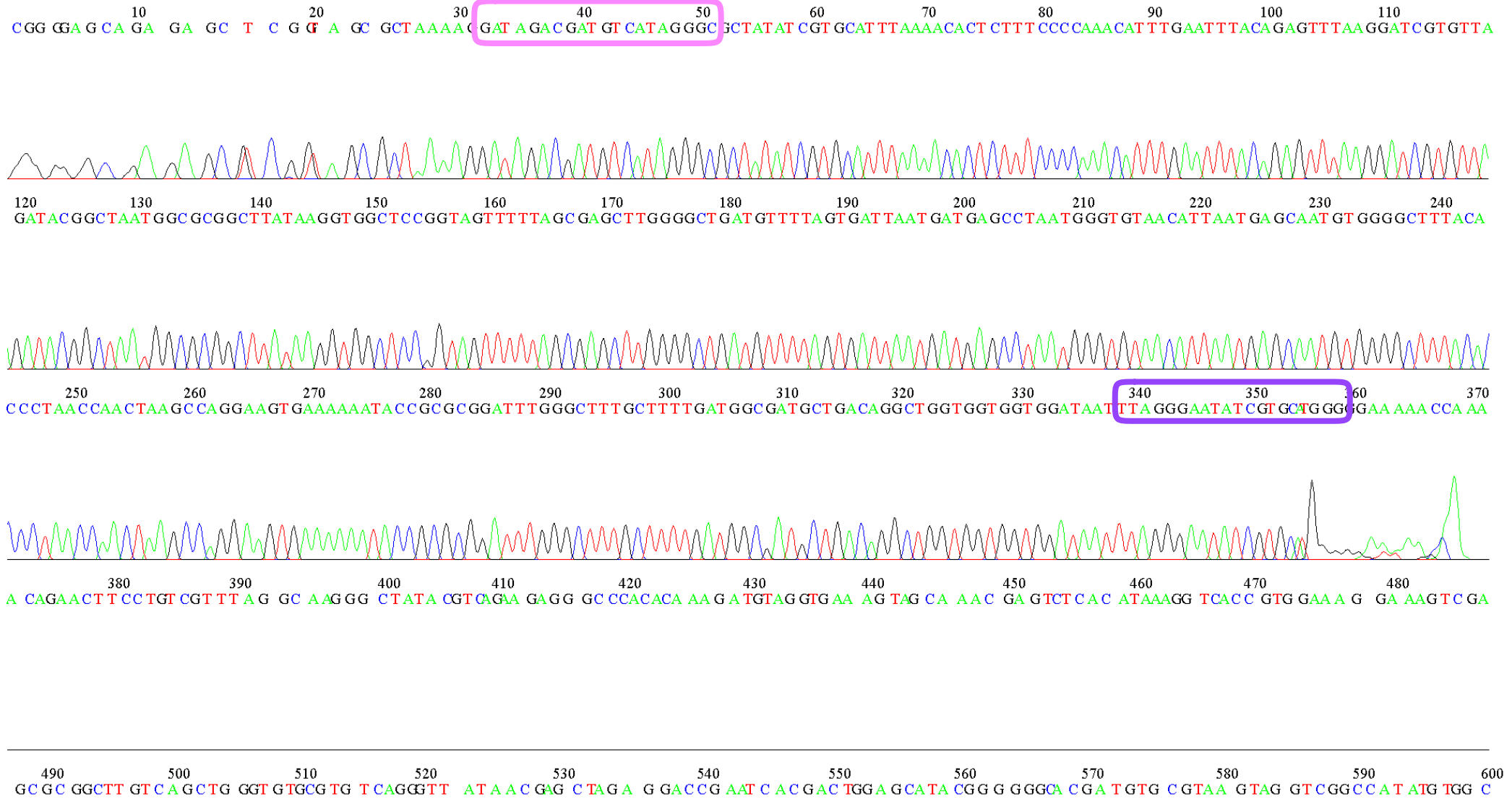

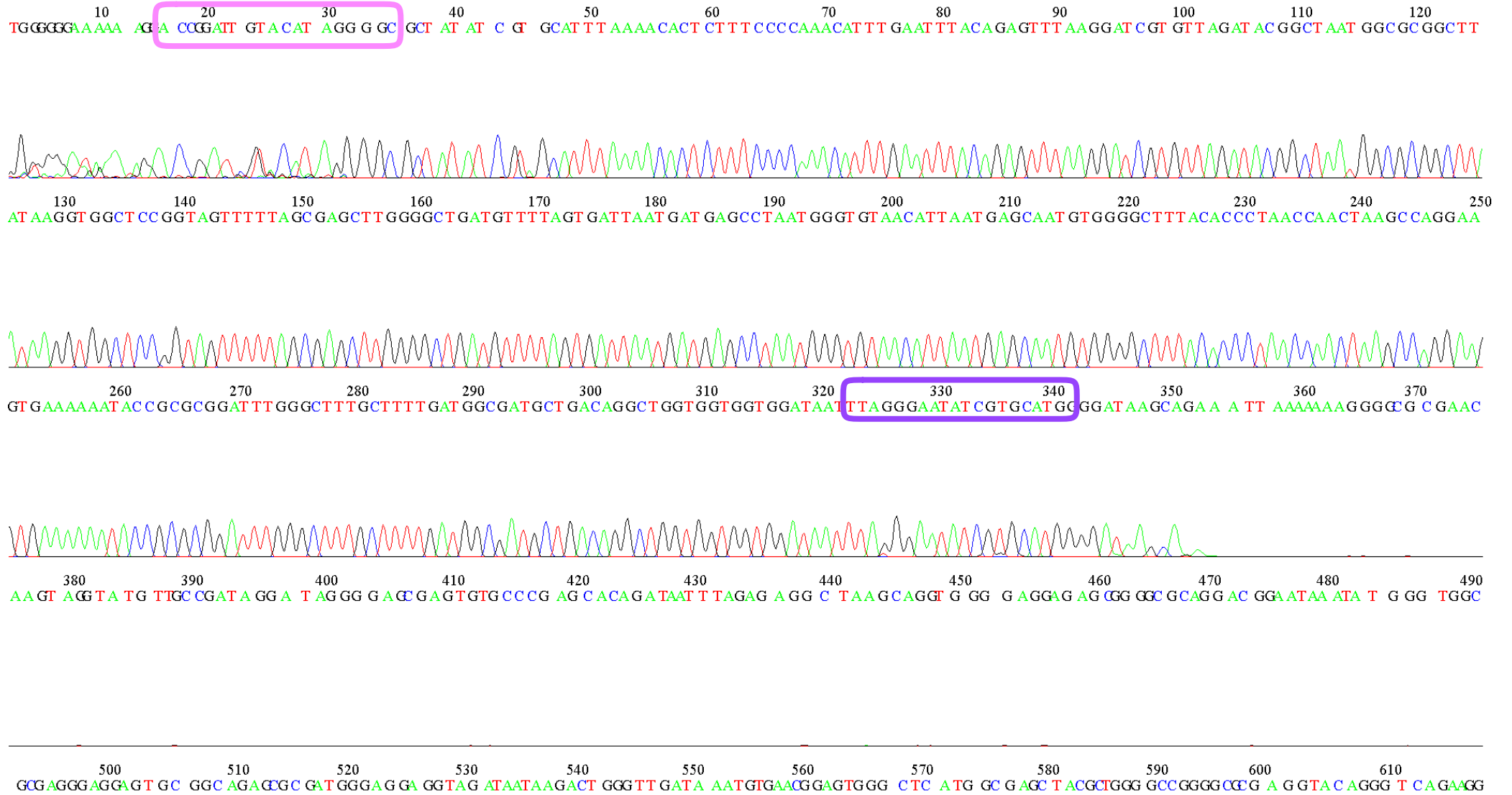

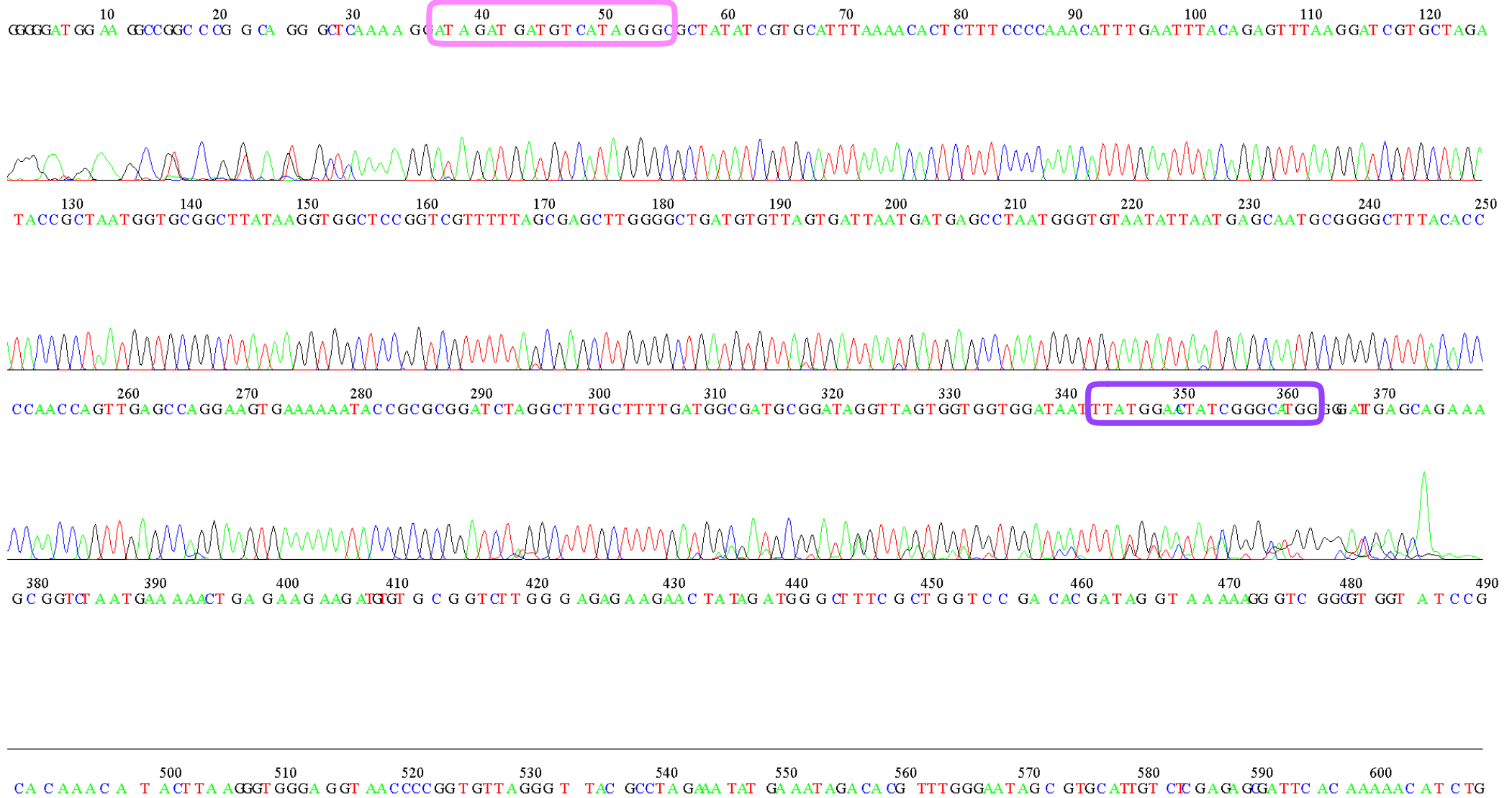

Supplement: Supplementary file 1 [file biomedicines-13-00914-s001.zip › Source S1.pdf]
